# Modelling the network of cell cycle transcription factors in the yeast *Saccharomyces cerevisiae*

**DOI:** 10.1186/1471-2105-7-381

**Published:** 2006-08-16

**Authors:** Shawn Cokus, Sherri Rose, David Haynor, Niels Grønbech-Jensen, Matteo Pellegrini

**Affiliations:** 1 Department of Molecular, Cell, and Developmental Biology, University of California, Los Angeles, USA; 2 Department of Biostatistics, University of California, Berkeley, CA, USA; 3 Department of Radiology, University of Washington, WA, USA; 4 Department of Applied Science, University of California, Davis, USA

## Abstract

**Background:**

Reverse-engineering regulatory networks is one of the central challenges for computational biology. Many techniques have been developed to accomplish this by utilizing transcription factor binding data in conjunction with expression data. Of these approaches, several have focused on the reconstruction of the cell cycle regulatory network of *Saccharomyces cerevisiae*. The emphasis of these studies has been to model the relationships between transcription factors and their target genes. In contrast, here we focus on reverse-engineering the network of relationships among transcription factors that regulate the cell cycle in *S. cerevisiae*.

**Results:**

We have developed a technique to reverse-engineer networks of the time-dependent activities of transcription factors that regulate the cell cycle in *S. cerevisiae*. The model utilizes linear regression to first estimate the activities of transcription factors from expression time series and genome-wide transcription factor binding data. We then use least squares to construct a model of the time evolution of the activities. We validate our approach in two ways: by demonstrating that it accurately models expression data and by demonstrating that our reconstructed model is similar to previously-published models of transcriptional regulation of the cell cycle.

**Conclusion:**

Our regression-based approach allows us to build a general model of transcriptional regulation of the yeast cell cycle that includes additional factors and couplings not reported in previously-published models. Our model could serve as a starting point for targeted experiments that test the predicted interactions. In the future, we plan to apply our technique to reverse-engineer other systems where both genome-wide time series expression data and transcription factor binding data are available.

## Background

Reverse-engineering networks of transcriptional regulation is a major goal of biology [1-6]. In the past few years, dramatic progress has been made in this effort due to significant improvements in experimental methodologies. Using chromatin immunoprecipitation in conjunction with microarrays, it is now possible to measure the binding of many transcription factors to the promoters of most genes, thus elucidating the underlying structure of the transcriptional regulatory network. For example, in the case of the yeast *Saccharomyces cerevisiae*, the binding profiles of 204 transcription factors to all promoters have been measured in rich media [7,8].

Despite the significant advance in our understanding that this data permits, it leaves a number of important questions unanswered. Does the binding of a specific transcription factor to the promoter of a gene regulate its expression? Does the transcription factor act alone or in cooperation with other factors? Under which conditions is a particular transcription factor active? In general, how does the connectivity of the network change in different conditions (e.g., during the cell cycle)?

To answer these questions, one must analyze the transcription factor binding data in conjunction with expression data collected from multiple conditions. Expression data is typically obtained using microarrays that allow monitoring of changes in the transcription of all mRNAs between samples. A variety of methods have been developed that utilize both expression data and transcription factor binding data to model transcriptional regulation. The simplest models identify transcription factor binding sites (or *cis *regulatory motifs) that are over-represented in the promoters of differentially-expressed genes [9]. Another approach estimates which transcription factors are globally active in a particular expression profile using multiple linear regression [5,10,11]. A more complex approach models the coupling strengths between transcription factors and their targets using Network Component Analysis [12].

These approaches usually work with the logarithm of the expression and binding data, and typically assume a linear or affine relationship between the two. However, it is likely that transcription factor binding affects gene expression in a nonlinear fashion, e.g., below some level it has no effect and above some higher level the effect might saturate. This type of behavior can be modelled using linear splines [13] or sigmoidal functions [14,15]. Moreover, there is abundant evidence that cooperative effects among transcription factors also play a critical role in regulating gene expression. As a result, a variety of approaches have been developed to identify non-linear cooperative effects between factors [13,16-19].

These methods allow one to identify biologically-meaningful couplings between transcription factors and their target genes in specific conditions. Other approaches attempt to model time-dependent changes in the transcriptional regulatory network. The dynamics of transcriptional regulation are usually studied during the cell cycle because time courses of gene expression of synchronized yeast cells have been measured [20,21]. Various approaches, including stochastic differential equations [14] and maximum likelihood-based methods [15], have been used to parameterize models of transcriptional regulation during the yeast cell cycle. Other groups have used experimental data to parameterize differential equations that describe a model of the yeast cell cycle [22].

All of these previous studies have focused on modelling the interactions between transcription factors and their target genes. In contrast, here we focus on the regulatory network of interactions among transcription factors themselves – such interactions play a central role in regulating cellular programs. Thus, we set out to construct a dynamical model of the couplings between the activities of transcription factors, hoping to elucidate the dynamics of this module. As in previous studies, we focus on the cell cycle of *S. cerevisiae *due to the availability of genome-wide time series expression data. The resulting model gives a view of the temporal program of transcriptional regulation for the key cell cycle regulators, thus defining the basic machinery that regulates this fundamental process.

**Figure 1:**
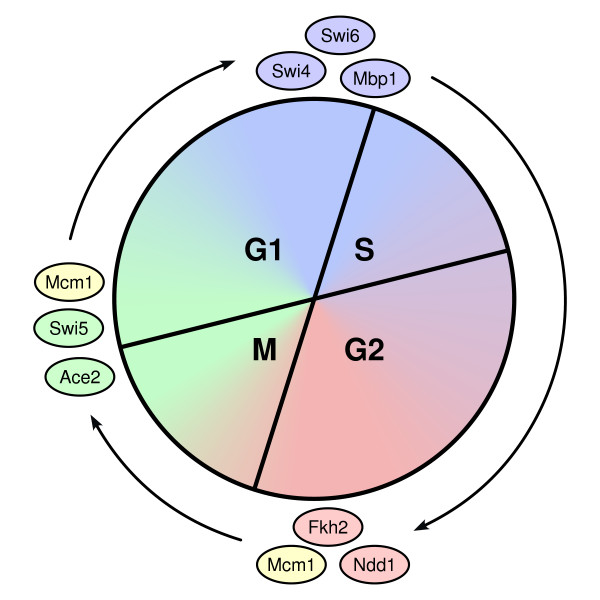
The canonical model of cell cycle regulation. The canonical model is determined primarily from transcription factor binding data. It contains 8 transcription factors that comprise 3 complexes that activate each other in a serial fashion to regulate the expression of genes during the cell cycle.

Previous studies have attempted to reconstruct the network of transcription factors that regulate the cell cycle primarily using transcription factor binding data [23,24]. Other studies have utilized expression and protein-protein interaction data to study the dynamics of transcription factor complex formation during the cell cycle [25]. These studies were able to show that the primary regulators of the cell cycle – the *canonical transcription factors *– are the Swi4-Swi6-Mbp1, Fkh2-Ndd1-Mcm1, and Mcm1-Swi5-Ace2 complexes. These regulate each other in a serial fashion: the transcription factors that control one phase of the cycle activate those that control the next phase (see Figure 1). The underlying assumption of this model – the *canonical model *– is that if a transcription factor binds to the promoter of another factor, it likely regulates its expression. However, as we have discussed above, these models of transcription factor binding do not offer a complete picture of transcriptional regulation since they do not explicitly predict changes in gene expression. To obtain a more complete understanding of transcriptional regulation, one must construct a dynamical model that accounts for expression data in terms of binding data. Here, we construct such a model and compare it to the canonical model.

For the sake of simplicity, we use a linear regression approach to estimate transcription factor activities. Our approach is similar to that used in Motif Regressor [5] and REDUCE [26] except that we are fitting expression data with transcription factor binding data instead of measurements of the presence of *cis *regulatory sequences in promoters. The "activity" of a transcription factor is measured by computing an α-*coefficient*, which is the regression coefficient of that factor in our linear model (see Methods below). The α-coefficient describes whether the genes a transcription factor is bound to are differentially expressed or not were the effects of other factors to be held constant. Factors with α > 0 are generally bound to the promoters of over-expressed genes while factors with α < 0 are binding to the promoters of under-expressed genes. If a transcription factor is an activator, then α > 0 implies that the factor is active and α < 0 implies that it is inactive. For repressors, the interpretation is the opposite: α > 0 and α < 0 correspond to the repressing transcription factor being inactive and active, respectively.

When these calculations are applied to a time series of expression profiles, we obtain the α-coefficients ("activities") of each transcription factor over time. Here, we are interested in the changes in transcription factor α-coefficients during the cell cycle of *S. cerevisiae*. Yeast cells were synchronized using yeast mating pheromone and the expression of all genes were profiled over two periods of the cell cycle [20]. From this data, we were able to not only compute the α-coefficients of the canonical cell-cycle-regulating transcription factors, but also able to identify additional factors that may play a significant role in regulation of the cell cycle.

We then investigated whether it was possible to model the temporal couplings of these factors in the form of ordinary differential equations (or difference equations, as expression data was only available at a small number of equally-spaced discrete times). Such a model would enable us to predict which factors are affecting each other's α-coefficients across the different phases of the cell cycle. We were able to construct such a model by generating a time-translation matrix of transcription factor α-coefficients using least squares [27]. The significant couplings in this model may be displayed in the form of a network and this network can be compared to the canonical model of transcriptional regulation of the yeast cell cycle. Such a comparison demonstrates that our model is consistent with the canonical model. At the same time, the new model provides a more comprehensive view of transcriptional regulation that includes additional factors and couplings.

## Results

We set out to reverse-engineer the interactions of the transcription factors comprising the core module of regulators of the *S. cerevisiae *cell cycle. The model captures the time-dependent α-coefficients of transcription factors and how they are coupled to control this important cellular program. The first step in our approach is to determine the α-coefficients of transcription factors during the cell cycle and to identify the factors that are the most significant regulators of the cell cycle.

We assume that changes in expression of a specific gene depend on the product of the binding ratios of all the transcription factors that bind to its promoter, i.e.,

Ri=∏j=1Nbijαj     (Equation 1)
 MathType@MTEF@5@5@+=feaafiart1ev1aaatCvAUfKttLearuWrP9MDH5MBPbIqV92AaeXatLxBI9gBaebbnrfifHhDYfgasaacH8akY=wiFfYdH8Gipec8Eeeu0xXdbba9frFj0=OqFfea0dXdd9vqai=hGuQ8kuc9pgc9s8qqaq=dirpe0xb9q8qiLsFr0=vr0=vr0dc8meaabaqaciaacaGaaeqabaqabeGadaaakeaacqWGsbGudaWgaaWcbaGaemyAaKgabeaakiabg2da9maarahabaGaemOyai2aa0baaSqaaiabdMgaPjabdQgaQbqaaGGaciab=f7aHnaaBaaameaacqWGQbGAaeqaaaaaaSqaaiabdQgaQjabg2da9iabigdaXaqaaiabd6eaobqdcqGHpis1aOGaaCzcaiaaxMaadaqadiqaaiabbweafjabbghaXjabbwha1jabbggaHjabbsha0jabbMgaPjabb+gaVjabb6gaUjabbccaGiabbgdaXaGaayjkaiaawMcaaaaa@4DC8@

where *R*_*i *_is the ratio of the expression level of gene *i *in two conditions, the α-*coefficient *α_*j *_is a measure of the contribution of transcription factor *j*,* b*_*ij *_is a constant (equal to 1 when transcription factor *j *is unrelated to gene *i*) giving the coupling between gene *i *and factor *j*, and *N *is the number of transcription factors in the model. In practice, we work with logarithms of expression ratios and logarithms of binding profiles so that (see Methods) one may solve for the α_*j *_by multiple linear regression. As discussed earlier, α_*j *_is a surrogate for the activity of transcription factor *j *and positive values indicate an active activator or inactive repressor and negative values indicate an active repressor or inactive activator. Without prior knowledge of the nature of transcription factor *j*, we do not know from its α_*j *_if that factor is active or inactive; rather (and more of concern as regards gene expression), we know that it is tending to bind over-expressed (α_*j *_> 0) or under-expressed (α_*j *_< 0) genes. Of course, in some cases we do know whether a transcription factor is an activator or repressor and can therefore easily transform its α-coefficient to activity. Nonetheless, for the remainder of this manuscript we focus on α-coefficients and not activities.

We applied the above to model the α-coefficients of transcription factors during two periods of the yeast cell cycle. We concentrated on modelling the expression data that was obtained by using yeast mating pheromone to synchronize yeast cells, although we also present models based on data from synchronization using a temperature-sensitive cdc15 mutant and from elutriation [20]. The binding was measured for 204 transcription factors in rich media using chromatin immunoprecipitation [7]. We computed α-coefficients not only for the canonical transcription factors, but also for additional factors we identified to be significant in regulating the cell cycle. These additional factors were selected by determining the significance of the contribution of each coefficient in regressions. For each time point, we identified the most significant transcription factors based on *p*-value, and factors were rank-ordered based on the number of time points in which they were significant. We filtered out factors that did not show periodic behavior. We identified four additional transcription factors – Bas1, Spt2, Ste12, and Yox1 – that are likely significant regulators of the cell cycle; stopping at four additional factors maintains a reasonable data-to-parameter ratio in the model. Details of the procedure used appear in the Methods section.

Having selected significant transcription factors and found their α-coefficients at each time point in the cell cycle, the second step in our approach is to model the system's dynamics. We accomplished this by computing a *transition *(or *time-translation*) *matrix T *that can be used to determine the α-coefficients of transcription factors at other time points from the α-coefficients at the current time point by matrix multiplication. Estimated using least squares, the matrix *T *permits inference of the network of couplings between transcription factors (see Methods).

We chose to perform constrained least squares and require the estimate of *T *to have only non-negative entries. This choice was made for several reasons. First, it generates models that are more readily interpretable biologically. It is clear that a positive entry may be interpreted as the positive α-coefficient of transcription factor *A *increasing the α-coefficient of transcription factor *B *at the next time point, as one might expect for activators. A positive entry may also be seen as the negative α-coefficient of transcription factor *A *decreasing the α-coefficient of transcription factor *B *at the next time point, as one might expect for repressors. In contrast, a negative entry suggests that the negative α-coefficient of a factor increases the α-coefficient of another, or vice-versa, which we expect to be less likely in 7-minute intervals for both activators and repressors. A second reason to apply non-negativity constraints to the entries of the time-translation matrix is that an unconstrained matrix tends to have no zero entries (i.e., all entries non-zero). This leads to an undesirable model in which all transcription factors depend on all other factors, which we know not to be true. Finally, applying constraints to the time-translation matrix can reduce the number of parameters needed to be fit and improve the model's data-to-parameter ratio.

We compared our model to the canonical model of the network of cell-cycle-regulating transcription factors (Figure 1) that has been inferred primarily from binding data by Simon, et al. [23,24]. Three groups of transcription factors – Swi4-Swi6-Mbp1, Fkh2-Ndd1-Mcm1, and Mcm1-Swi5-Ace2 – are known to be active in three phases of the cell cycle (G1/S, G2/M, and M/G1, respectively), binding to each other in a serial fashion: the first group binds to promoters of the second, the second to the third, and the third to the first. This basic mechanism serves to regulate the transcription of genes during the cell cycle.

**Figure 2:**
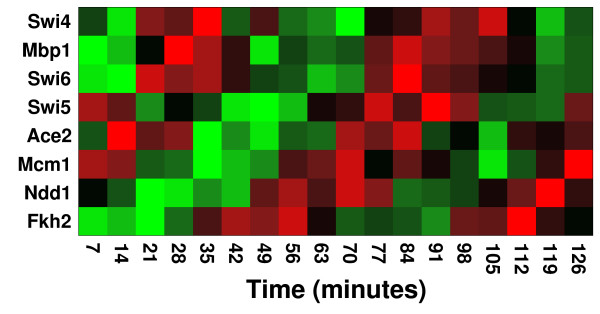
α-coefficients of transcription factors regulating the cell cycle. A heat map of the α-coefficients of the canonical transcription factors during two periods of the cell cycle. Red indicates α > 0 (transcription factor generally bound to over-expressed genes), green indicates α < 0 (transcription factor generally bound to under-expressed genes), and black indicates α = 0. Factors are ordered based on their phase in the long-term asymptotic behavior of our dynamical model. The α-coefficients have been quantile normalized for each factor across the time points.

**Figure 3:**
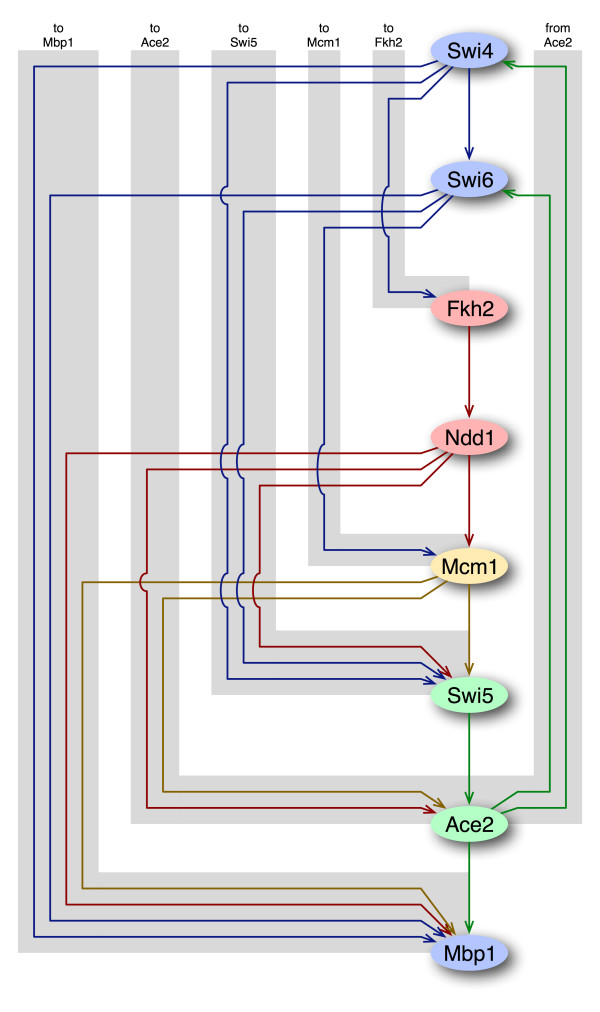
Transcription factor network of canonical cell cycle regulators. We depict the network of canonical transcription factors derived using our approach. We construct a dynamical model of their α-coefficients and show the non-zero edges in this matrix as directed links between factors. A line from factor *A *to factor *B *indicates that the activity of *A *affects the activity of *B *at the next time point. The displayed order of factors minimizes the number of upward arcs (these arcs being grouped on the right side of the figure).

To compare this model to the one generated using our approach, we started with the 8 canonical transcription factors. We computed the α-coefficients of these transcription factors in the cell cycle time course data (see Figure 2) and then computed the transition matrix that describes their dynamics. This matrix may be viewed as a network, seen in Figure 3. (Note that if two transcription factors are connected in a model by arcs in both directions, we display only the more significant direction.) In Additional files 1 and 2, we show the same network computed using two other time courses in the Spellman, et al. data set. Additional file 1 shows the network derived from the cdc15 synchronization experiments and Additional file 2 displays the network derived from the elutriation time series. In all three networks, we see the same central relationship Fkh2 → Ndd1 → Mcm1 → Ace2 → Swi4/Swi6/Mbp1 between transcription factors, demonstrating the robustness of our approach in reconstructing the core regulatory cycle independently of the synchronization method used to generate the expression data.

The resulting models are quite similar to the canonical one. They show a flow of influence from Ace2, a component of the M/G1 complex, to the Swi4-Swi6-Mbp1 complex active in G1/S. The Swi4 subunit of this complex influences the α-coefficient of Fkh2, which in turn affects Ndd1 and subsequently Mcm1, both subunits of the G2/M complex. The cycle is then completed with interactions from Ndd1 and Mcm1 back to Ace2 and Swi5. All of these interactions recapitulate the basic flow of the canonical model indicating strong support for this model using our approach. Nonetheless, we note that although 8 of the 16 edges in Simon, et al.'s model [23] are recapitulated in our 25-arc model derived from the pheromone-synchronized data, the resulting *p*-value for the overlap of arcs between the two models is not statistically significant. We believe that this is due in part to limitations inherent in Simon's model which captures only binding data and omits influences of the activity of one factor on another which may be mediated by mechanisms other than by the binding of a transcription factor to the promoter of another. The arcs missing from our model and present in Simon's may be explained as well. If multiple transcription factors are activating transcription of another factor (as in Simon's model), our model may select only a single one of these to best fit the data.

We next built a more general model by identifying in an unsupervised fashion which additional transcription factors most significantly regulate the cell cycle. As explained in the Methods section, for each of the 204 transcription factors we measure not only its α-coefficient at each time point, but also the significance of this estimate in the form of a *p*-value. We used an iterative approach, keeping only transcription factors that had a *p*-value below 0.1, re-computing the α-coefficients and *p*-values for the reduced set of transcription factors. We retained the factors that were significant in the largest number of time points. Among these, we selected those that had periodic α-coefficients. Periodicity was determined using a discrete Fourier transform to compute the fraction of the power spectrum of the α-coefficients of a given transcription factor that laid within a range consistent with the measured time period of 1 cell cycle of *S. cerevisiae *(see Methods).

**Figure 4:**
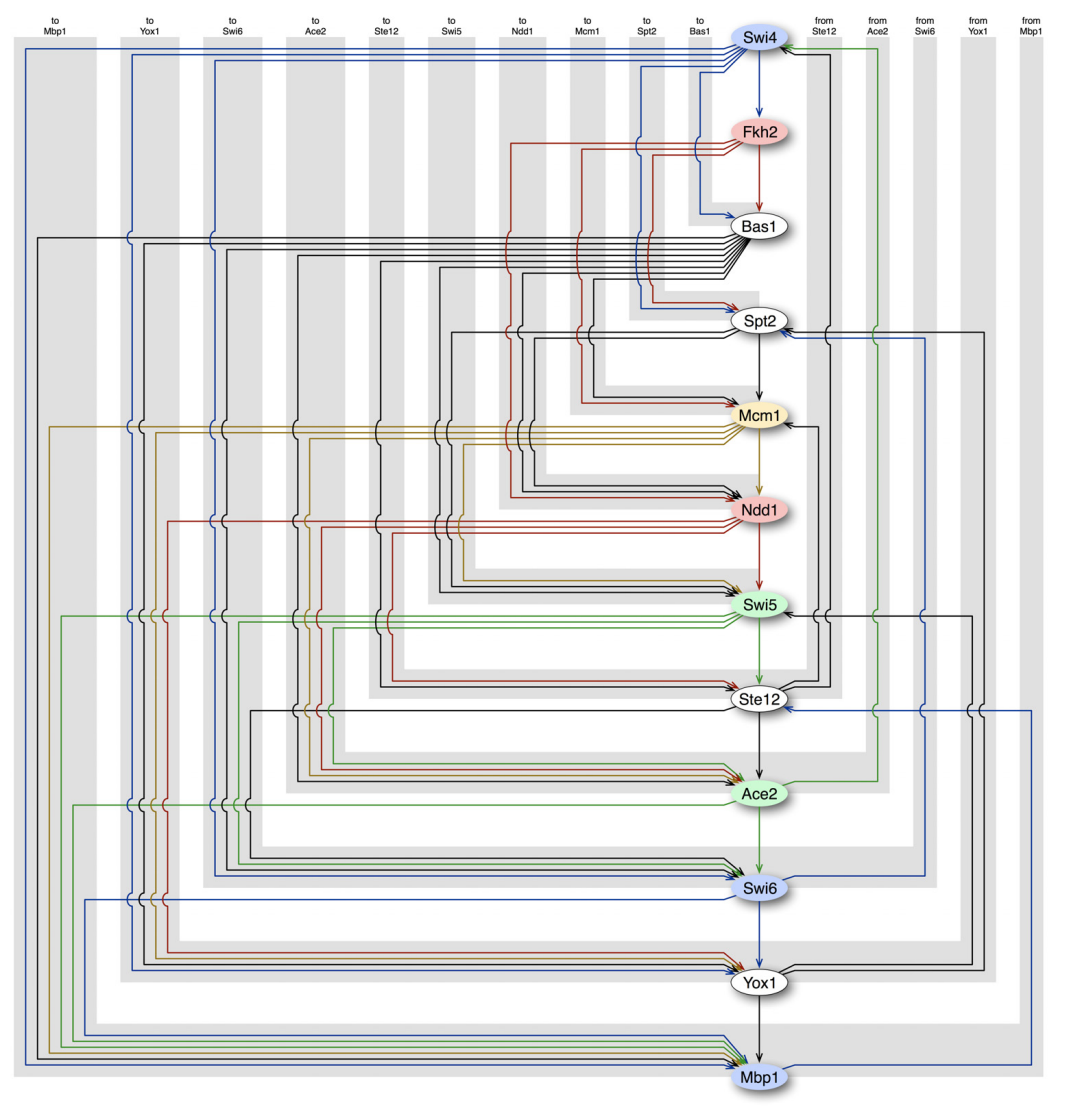
Full network of transcription factors regulating the cell cycle. We identified the 4 transcription factors that were most significantly regulating the cell cycle and considered them along with the 8 canonical factors. We constructed a dynamical model of their α-coefficients and show here the non-zero entries in this matrix as directed links between the 12 factors. The resulting network represents the complete network of couplings between cell cycle regulating transcription factors. A line from factor *A *to factor *B *indicates that the activity of *A *affects the activity of *B *at the next time point. The displayed order of factors minimizes the number of upward arcs (these arcs being grouped on the right side of the figure).

As a result, we selected four additional transcription factors – Bas1, Spt2, Ste12, and Yox1 – that significantly regulate the cell cycle. Their α-coefficients are shown in Additional file 3. As shown in Table I, most of these have some supporting evidence that they are involved in cell cycle regulation. The exception is Bas1, a factor involved in the purine and histidine pathways that has no known connection to the cell cycle. The robustness of our unsupervised selection methodology is evident by its recovery of 6 of the 8 canonical transcription factors among the top 10 factors (and the probability of seeing such a significant overlap by chance is less than 10^-3^). Finally, we computed the transition matrix for the 12 factors (the 8 canonical and 4 additional factors). This gives our most complete model of transcription factor couplings in the yeast cell cycle (see Figure 4).

**Figure 5:**
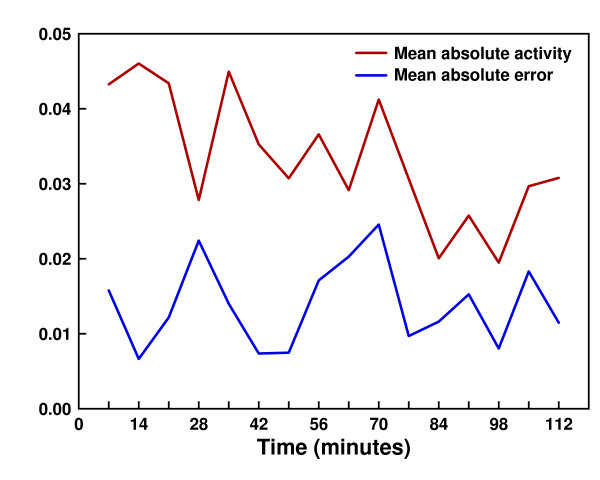
Model residuals. Shown is a comparison of the α-coefficients of transcription factors estimated using regression and displayed in Additional file 3 to those predicted by our dynamical model. We plot the mean absolute value of the α-coefficients across two periods of the cell cycle versus the mean absolute value of the residuals (i.e., the difference between the measured and predicted α-coefficients). Each time step represents a 7-minute interval. We see that the model has significant predictive power, although this diminishes as the cell cycle progresses. This may be due in part to gradual loss of synchrony over time in the experimental yeast cells from which expression was measured.

Although the model with 12 transcription factors is significantly more complex than the network of canonical transcription factors, it recapitulates much of the basic flow observed earlier. For example, we still see the flow of influence from Swi4 to Fkh2 to Ndd1 to Swi5 and back to Swi6. Furthermore, we see additional interactions that are supported by experimental evidence. For instance, the edge between Ste12 and Mcm1 is supported by experimental evidence that Ste12 interacts physically with Mcm1 [28]. Similarly, the link between Mcm1 and Yox1 is supported by experimental evidence that these two interact [29]. We show the mean absolute error (i.e., average magnitude of residuals) in the 12-factor model in Figure 5. The model has significant predictive power, although (not unexpectedly) this diminishes as the cell cycle progresses; the residuals are an increasing fraction of mean absolute activity as time increases. This is consistent with the experimental yeast cells gradually losing synchrony in the real data but not in the model.

We performed an analysis of the long-term dynamical properties of the model. As explained in detail in the Methods section, an eigensystem decomposition of the model allows us to identify its modes. In particular, we are interested in the modes whose complex eigenvalues are of magnitude closest to unity, since these modes give the long-term cyclical behavior of the system. Modes whose eigenvalues have magnitude less than 1 die off exponentially over time. Our model also has a single real eigenvalue greater than 1. The mode associated with this eigenvalue captures the fact that α-coefficients of the transcription factors grow over multiple cycles.

**Figure 6:**
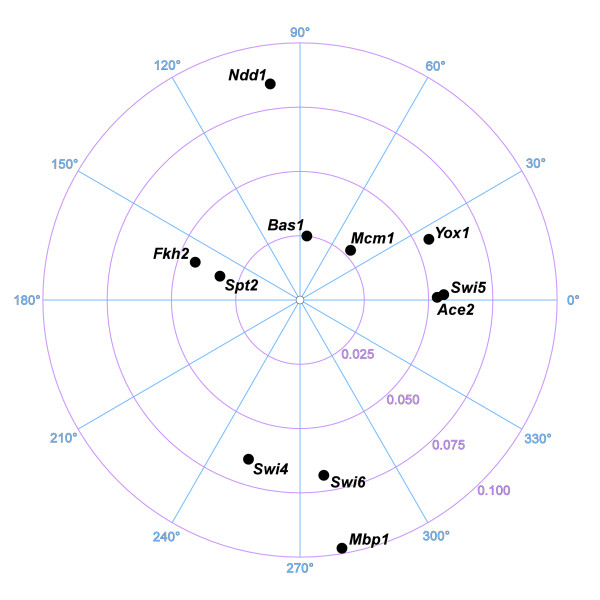
Asymptotic magnitudes and phases of transcription factor α-coefficients. We perform a dynamical analysis of our 7-minute time-translation matrix to identify the mode that corresponds to the long-term oscillatory behavior of our system. For this mode, we compute the amplitudes and phases that describe the α-coefficients of each factor. The cell cycle proceeds clockwise. Relative to Figure 1, this figure is rotated by approximately 180°.

The modes from the eigenvalues of magnitude closest to unity generate periodic behavior with a period of 60 minutes, which is very close to the 63-minute period observed in the Spellman, et al. data. Furthermore, we can estimate the long-term (i.e., asymptotic) behavior of each factor, which we describe in terms of amplitude and phase (see Methods) and report in Table I and Figure 6. We see that the order of the phases of each factor corresponds closely to those expected from the canonical model: Swi5 and Ace2 are around 0°, Swi4, Swi6, and Mbp1 are around 270°, and Ndd1 is close to 90°. In contrast to the canonical model, however, we see that the α-coefficient of Fkh2 peaks before that of Ndd1. We note that the α-coefficient of Mcm1 peaks between that of Ndd1 and those of Swi5 and Ace2, which is not surprising since it is involved both in the G2/M and M/G1 complexes. We also see from our analysis that Mbp1 and Ndd1 appear to have the highest amplitudes during the course of the cell cycle, while Bas1 – the factor that appears to have no other evidence of its involvement in the cell cycle – has the smallest amplitude.

## Discussion

Many cellular programs, such as control of the cell cycle or the metabolic cycle [30], are regulated through the temporally-patterned activation of specific transcription factors. In order to understand these programs more completely, it is necessary to build dynamical models of transcriptional regulation. Approaches to the construction of these models often involve either the collection of coupling parameters between molecules by direct experimental measurements or estimation of model parameters from high-throughput data.

Model building using the former approach by using sets of individual molecular coupling measurements can severely limit the scope of the model. Such measurements are often only available for a small number of molecules. As a result of this limitation, most of these types of models capture couplings between proteins and not between transcription factors and their target genes due to the larger number of parameters such would involve. Furthermore, these types of models are difficult to validate since they cannot be compared to systematically-collected comprehensive time series data covering all model variables.

A previous dynamical model [22] of the yeast cell cycle following the direct-measurement paradigm included about a dozen molecules that are known to be involved in regulating the cell cycle (e.g., clb2, clb5, cln2, and cdc14), including the transcription factors SBF (composed of Swi4 and Swi6), MBF (composed of Swi6 and Mbp1), and Mcm1. The more than 100 parameters that are involved in this model were estimated from individual experiments and so only a very limited number of couplings between the transcription factors in the model and their targets were included. One appealing characteristic, however, is that this model allows one to explicitly predict the dynamics of mutant strains that lack individual components of the model. In fact, the authors were able to demonstrate that their model reproduced the phenotypes of many cell-cycle-deficient mutants. However, these comparisons are inevitably more qualitative than quantitative and can only be performed for mutations of modelled genes.

Utilizing the second paradigm, various methodologies have been developed to reconstruct the regulatory network of the yeast cell cycle from high-throughput data [14,15]. These approaches allow systematic comparison of model predictions to genome-wide data and they are not restricted *a priori *to include only a small subset of molecules. However, since these models only account for the regulation of genes by transcription factors, they have not been used to predict the phenotype of cell cycle mutants as such phenotypes do not usually involve mutations in transcriptional regulation.

In contrast to both of these approaches, we have presented a methodology for constructing dynamic models of the core modules of transcriptional regulation from high-throughput data sets. Our model describes the time-dependent α-coefficients of transcription factors during the cell cycle. As described above, the model was estimated using both expression and transcription factor binding data. As with other cell cycle models derived from high-throughput data, we are able to directly measure the fit of our model with respect to the data (e.g., as in Figure 5). Our approach need not make any *a priori *assumptions about which proteins should be included as these may be determined directly from the data.

Using our model, we are able to incorporate the effect of transcription factors binding to all of their known targets rather than just a select subset and demonstrate that couplings between transcription factors are similar to those found in the canonical models of cell cycle regulation. These couplings permit the activity of a transcription factor to affect the activity of another by, for example, activating its transcription, as well as allowing for the possibility that two factors act cooperatively to turn on genes. The underlying principle of our model (e.g., as seen in Equation 1) is that all factors act cooperatively at each promoter at some level. The α-coefficient of a given factor at a given time point is a measure of the extent of the global influence that factor has at that time. Cooperation between factors in our model is therefore manifested by correlation in the α-coefficients of two factors. For example, in Figure 2, it is clear that Mbp1 and Swi6 have correlated α-coefficients and they are indeed known to act in a cooperative fashion since they form the MBF complex. The link between these factors in our networks is capturing cooperative activity (and not transcriptional activation of one factor by another).

In conclusion, unlike other genome-wide models of cell cycle transcription, our approach focuses on the relationships between transcription factors and not on the identification of the regulators of individual genes. Since transcription factors are the key regulators of transcriptional programs, we believe this approach provides a more comprehensive view of how these processes are regulated temporally. Furthermore, although it is not possible to completely validate the resulting networks, the results are reasonable from a biological perspective and largely conform to previous models of transcriptional regulation of the yeast cell cycle. As a result, our methodology should reasonably reconstruct regulatory networks whenever expression time series and transcription factor binding data are both available.

## Conclusion

We have presented a methodology for describing the temporal regulation of the yeast cell cycle. Our approach allows us to first identify the key regulators of this process, recovering most of the known regulators as well as new ones for which there is some supporting evidence for their involvement. More importantly, our methodology allows us to synthesize the manner in which these transcription factors coordinately regulate the cell cycle. This approach goes beyond existing static models of transcriptional factor networks involved in cell cycle regulation by demonstrating how the estimated transcriptional activities of these proteins are coupled in time.

We believe the methodology we have presented may be applied to model many different biological phenomena. In the case of *Saccharomyces cerevisiae*, time series expression data of a variety of cellular programs (e.g., the diauxic shift [31] and the metabolic cycle [30]) are already available. In higher eukaryotes, high-throughput data measuring transcriptional changes during circadian cycles has been measured (e.g., [32]). The availability of this and other data presents us a unique opportunity to model the key modules of the associated transcriptional networks, shedding new light on how cells control these complex phenomena.

## Methods

### Estimation of transcription factor α-coefficients

Taking a logarithm of both sides of Equation 1, we model the logarithm of the ratio of expression data as a linear combination of the logarithms of the transcription factor binding data:

log⁡(Ri)=∑j=1Nαjlog⁡(bij).
 MathType@MTEF@5@5@+=feaafiart1ev1aaatCvAUfKttLearuWrP9MDH5MBPbIqV92AaeXatLxBI9gBaebbnrfifHhDYfgasaacH8akY=wiFfYdH8Gipec8Eeeu0xXdbba9frFj0=OqFfea0dXdd9vqai=hGuQ8kuc9pgc9s8qqaq=dirpe0xb9q8qiLsFr0=vr0=vr0dc8meaabaqaciaacaGaaeqabaqabeGadaaakeaacyGGSbaBcqGGVbWBcqGGNbWzcqGGOaakcqWGsbGudaWgaaWcbaGaemyAaKgabeaakiabcMcaPiabg2da9maaqahabaacciGae8xSde2aaSbaaSqaaiabdQgaQbqabaaabaGaemOAaOMaeyypa0JaeGymaedabaGaemOta4eaniabggHiLdGccyGGSbaBcqGGVbWBcqGGNbWzcqGGOaakcqWGIbGydaWgaaWcbaGaemyAaKMaemOAaOgabeaakiabcMcaPiabc6caUaaa@4B17@

Here, *R*_*i *_is the ratio of expression of gene *i *to control, α_*j *_is the regression coefficient of transcription factor *j*, and *b*_*ij *_is the measured binding of transcription factor *j *to the promoter of gene *i*. We estimate the coefficients α_*j *_using multiple linear regression as implemented in the MATLAB function robustfit, a function that also returns an estimate of the standard error of each α_*j*_. The binding coefficients *b*_*ij *_are from Harbison, et al. [7] and we treat these as known parameters that are not to be fitted. The expression ratios *R*_*i *_are from Spellman, et al. [20].

We pre-filtered the binding data by eliminating all transcription factors that had more than 10 missing values across all genes, leaving 181 factors. We next eliminated all genes that had more than 3 missing values across the 18 time points, leaving 4,033 genes.

For each of the 181 transcription factors, robustfit computes the probability that each α_*j *_is non-zero. Roughly, a *t*-test is made for the ratio of each coefficient divided by its standard error. A detailed derivation of the *p*-value, which is beyond the scope of this manuscript, may be found in standard statistical texts [33,34]. We next use an iterative approach to select only transcription factors that have a *p*-value below 0.1. We first perform a regression step (initially with the full transcription factor binding matrix) and obtain a *p*-value for each of the α_*j*_. We then remove from the binding matrix *b*_*ij *_all columns that are associated with transcription factor α-coefficients whose *p*-values do not pass the threshold. This procedure is repeated with the reduced binding matrix until all transcription factor α-coefficients have *p*-values below 0.1. The choice of 0.1 as threshold is somewhat arbitrary, but this choice has only a minimal effect on the calculation; we have repeated the procedure with other *p*-value thresholds such as 0.01 and 0.001 and we obtain similar lists of significant factors and α-coefficients as seen in Additional file 4. We also note that the *p*-values for the final α-coefficients of the factors that are selected by the iterative procedure are very significant (*p *< 10^-10^) in all cases.

### Determination of time-translation matrices

We model the dynamics of the system by determining the transition matrix *T *that predicts the α-coefficients at the next time point from the α-coefficients at the current time point:

*A*(*t*+1) = *TA*(*t*).

The matrix *T *is computed using least squares constrained to produce only non-negative entries via the MATLAB function lsqnonneg. From this matrix, we can infer the network of couplings between transcription factors by identifying the most significant interactions. In cases where two factors were linked by arcs in both directions, we display only the more significant arc.

### Computation of asymptotic amplitudes and phases of transcription factor α-coefficients

To analyze the dynamics of the time-translation matrix, we compute an eigensystem of it using the MATLAB function eigen. Since we are interested in the long-term asymptotic behavior of our model, we focus on the two non-real conjugate eigenvalues whose magnitude is closest to unity and the two conjugate eigenvectors associated with them. The eigenvectors with eigenvalues smaller than these in magnitude decay exponentially in time compared to this mode and hence contribute nothing asymptotically. The spectrum of eigenvalues is shown in Additional file 5. The spectrum does contain a single real eigenvalue greater than 1. This generates growing α-coefficients, and is likely a result of the cyclical nature of the network with only non-negative coefficients, these generating positive feedback. However, for the purpose of estimating the relative phases and amplitudes of the transcription factor α-coefficients, it is appropriate to focus on the already-mentioned non-real eigenvalues of greatest magnitude.

Using the polar form

λ = |λ|*e*^*i*θ^

for the dominant non-real eigenvalue of the time-translation matrix in the upper half-plane, the period of the associated mode is given by

τ=2πΔtθ
 MathType@MTEF@5@5@+=feaafiart1ev1aaatCvAUfKttLearuWrP9MDH5MBPbIqV92AaeXatLxBI9gBaebbnrfifHhDYfgasaacH8akY=wiFfYdH8Gipec8Eeeu0xXdbba9frFj0=OqFfea0dXdd9vqai=hGuQ8kuc9pgc9s8qqaq=dirpe0xb9q8qiLsFr0=vr0=vr0dc8meaabaqaciaacaGaaeqabaqabeGadaaakeaaiiGacqWFepaDcqGH9aqpdaWcaaqaaiabikdaYiab=b8aWjabfs5aejabdsha0bqaaiab=H7aXbaaaaa@36C0@

where Δ*t *is the time (here, 7 minutes) between successive points (e.g., microarrays) in the expression time series.

Collect the eigenvectors of the time-translation matrix as the columns of a complex matrix *V *and let complex matrix *W *be the matrix inverse of *V*. To find the long-term asymptotic phase and amplitude of each transcription factor, let *z *be the complex number that is the dot product of the row of *W *corresponding to one of the dominant non-real eigenvalues with the initial state, and let *v *be the column of *V *corresponding to this same eigenvalue. Then the asymptotic amplitudes and phases of the transcription factors are the polar forms of the successive entries of 2*zv*. A detailed derivation is provided in Additional file 6.

### Identification of periodic transcription factors

To identify the transcription factors that have periodic α-coefficients, we compute the discrete Fourier transform of the α-coefficient profile for each factor. Taking the magnitudes of Fourier components (i.e., the "power spectrum") we calculate the fraction of spectral power that falls in components 7, 8, 9, and 10 as these correspond to an interval of periods containing the expected period for a single cycle of the Spellman data.

## Authors' contributions

The calculations described in this manuscript were performed in roughly equal contributions by SC, SR, and MP. DH and NGJ provided essential comments and guidance. The manuscript was written by MP and edited by SC. Network layouts, Figure and Additional file drawings, and Additional File 6 were by SC.

## Supplementary Material

Additional File 1 Transcription factor network of canonical cell cycle regulators as derived from the cdc15 time course. We show the non-zero entries in the model's time-translation matrix as directed arcs between transcription factors. We note the general similarity of the causal flow in this network to Figure 3 which was derived from the mating pheromone arrest time course. The order of factors displayed here minimizes the number of upward arcs (these arcs being grouped on the right side). Click here for file

Additional File 2 Transcription factor network of canonical cell cycle regulators as derived from the elutriation time course. We show the non-zero entries in the model's time-translation matrix as directed arcs between transcription factors. We note the general similarity of the causal flow in this network to Figure 3 which was derived from the mating pheromone arrest time course. The order of factors displayed here minimizes the number of upward arcs (these arcs being grouped on the right side). Click here for file

Additional File 3 α-coefficients of transcription factors regulating the cell cycle. A heat map (in the style of Figure 2) of the α-coefficients of the transcription factors from our extended 12-factor model during two periods of the cell cycle. Click here for file

Additional File 4 Transcription factor rankings used to select the 4 non-canonical transcription factors. Factors were ranked based on the number of time points in which their *p*-values were significant beyond some threshold (*p *< 0.1, 0.01, or 0.001). They were further ranked based on the periodicity of their α-coefficients. Canonical factors are shown in red, significant non-canonical factors are shown in yellow, and other factors are shown in white. We see that the same set of 5 non-canonical factors is selected as most significant for all of the *p*-value thresholds. Click here for file

Additional File 5 Eigenvalues of the time-translation matrix of the 12-factor model. Each application of the time-translation matrix moves time forward 7 minutes. Click here for file

Additional File 6 Asymptotic phases and amplitudes. A derivation of the formulas used for the asymptotic phases and amplitudes of components of real systems governed by a general class of real time-translation matrices is given. Click here for file

## Figures and Tables

**Table 1:** Predicted cell cycle regulators.

*TF*	*Phase*	*Amplitude*	*Periodicity*	*Notes*
**Ste12**	**318°**	**0.03**	**0.26**	**interacts with Mcm1**
Mbp1	280°	0.10	0.30	G1/S, member of the Swi6-Mbp1 (MBF) and Swi4-Swi6-Mbp1 complexes
Swi6	278°	0.07	0.32	G1/S, member of the Swi4-Swi6 (SBF), Swi6-Mbp1 (MBF), and Swi4-Swi6-Mbp1 complexes
Swi4	252°	0.07	0.33	G1/S, member of the Swi4-Swi6 (SBF) and Swi4-Swi6-Mbp1 complexes
**Spt2**	**164°**	**0.03**	**0.24**	**interacts with both histones and SWI-SNF components**
Fkh2	160°	0.04	0.26	G2-specific transcription in the mitotic cell cycle, forms complex with Ndd1 and Mcm1
Ndd1	098°	0.08	0.30	late S, forms complex with Fkh2 and Mcm1
**Bas1**	0**84°**	**0.02**	**0.21**	**regulation of basal and induced expression of genes of the purine and histidine biosynthesis pathways**
Mcm1	044°	0.03	0.27	DNA replication initiation (TAS), member of Fkh2-Ndd1-Mcm1 and Mcm1-Swi5-Ace2 complexes
**Yox1**	0**25°**	**0.06**	**0.19**	**binds to Mcm1p and Early Cell-cycle Boxes (ECBs) in the promoters of cell cycle-regulated genes expressed in M/G1**
Swi5	002°	0.06	0.20	G1 and at the M/G1 boundary, forms complex with Mcm1 and Ace2
Ace2	001°	0.05	0.29	G1, forms complex with Mcm1 and Swi5

Shown are the cell cycle regulators used in the expanded 12-factor model. The phases and amplitudes of the α-coefficients of each factor are calculated from the model's long-term asymptotic dynamics. Transcription factors are ordered by phase and those not in the canonical model are shown in bold.
