# Supplementary figures and images for: Modelling the network of cell cycle transcription factors in the yeast Saccharomyces cerevisiae
Source: BMC Bioinformatics. 2006 Aug 16;7:381. doi: 10.1186/1471-2105-7-381 (PMC1570153; doi:10.1186/1471-2105-7-381)

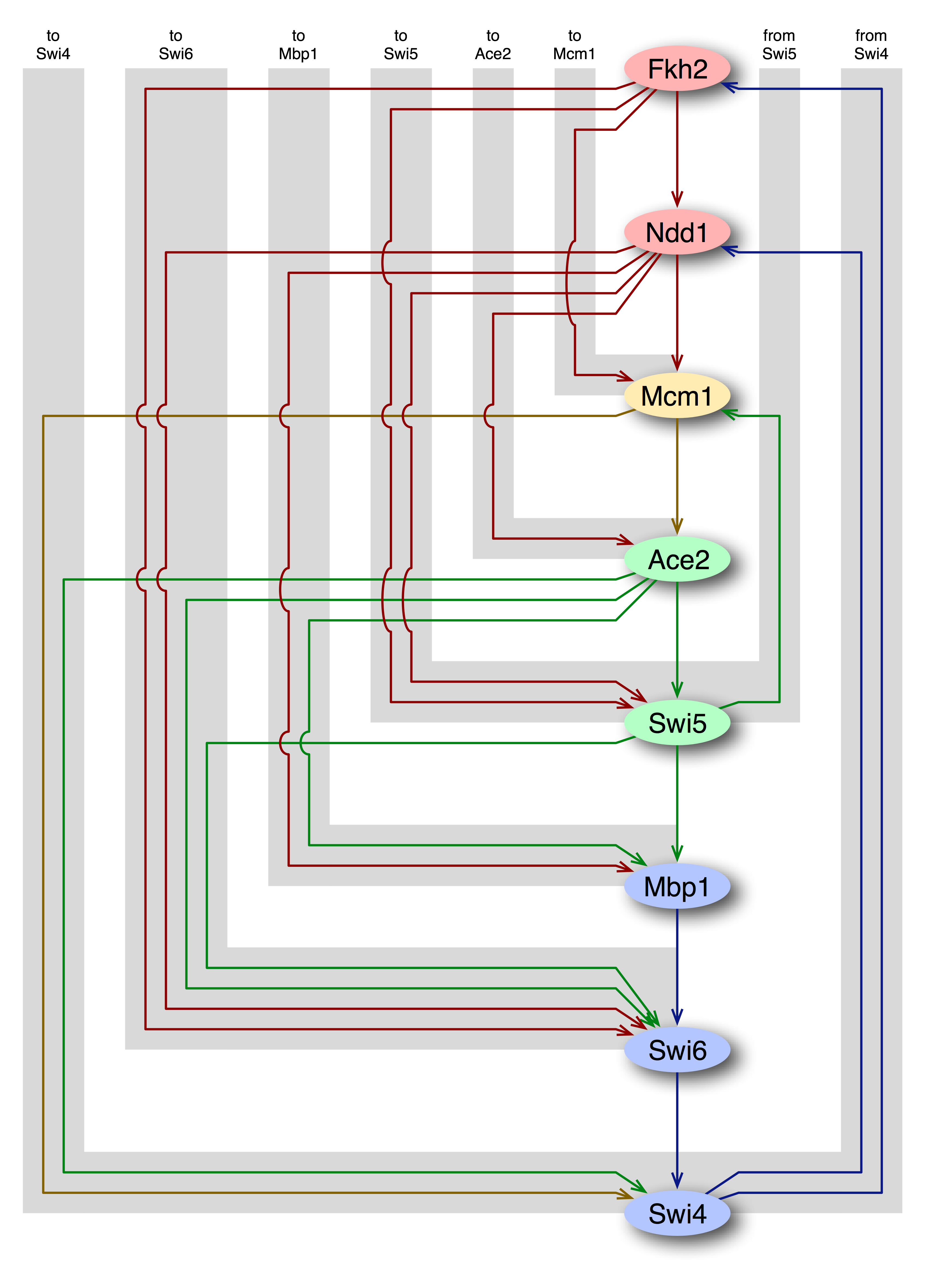

Supplement: Additional File 1 — Transcription factor network of canonical cell cycle regulators as derived from the cdc15 time course. We show the non-zero entries in the model's time-translation matrix as directed arcs between transcription factors. We note the general similarity of the causal flow in this network to Figure 3 which was derived from the mating pheromone arrest time course. The order of factors displayed here minimizes the number of upward arcs (these arcs being grouped on the right side). [file 1471-2105-7-381-S1.tiff]

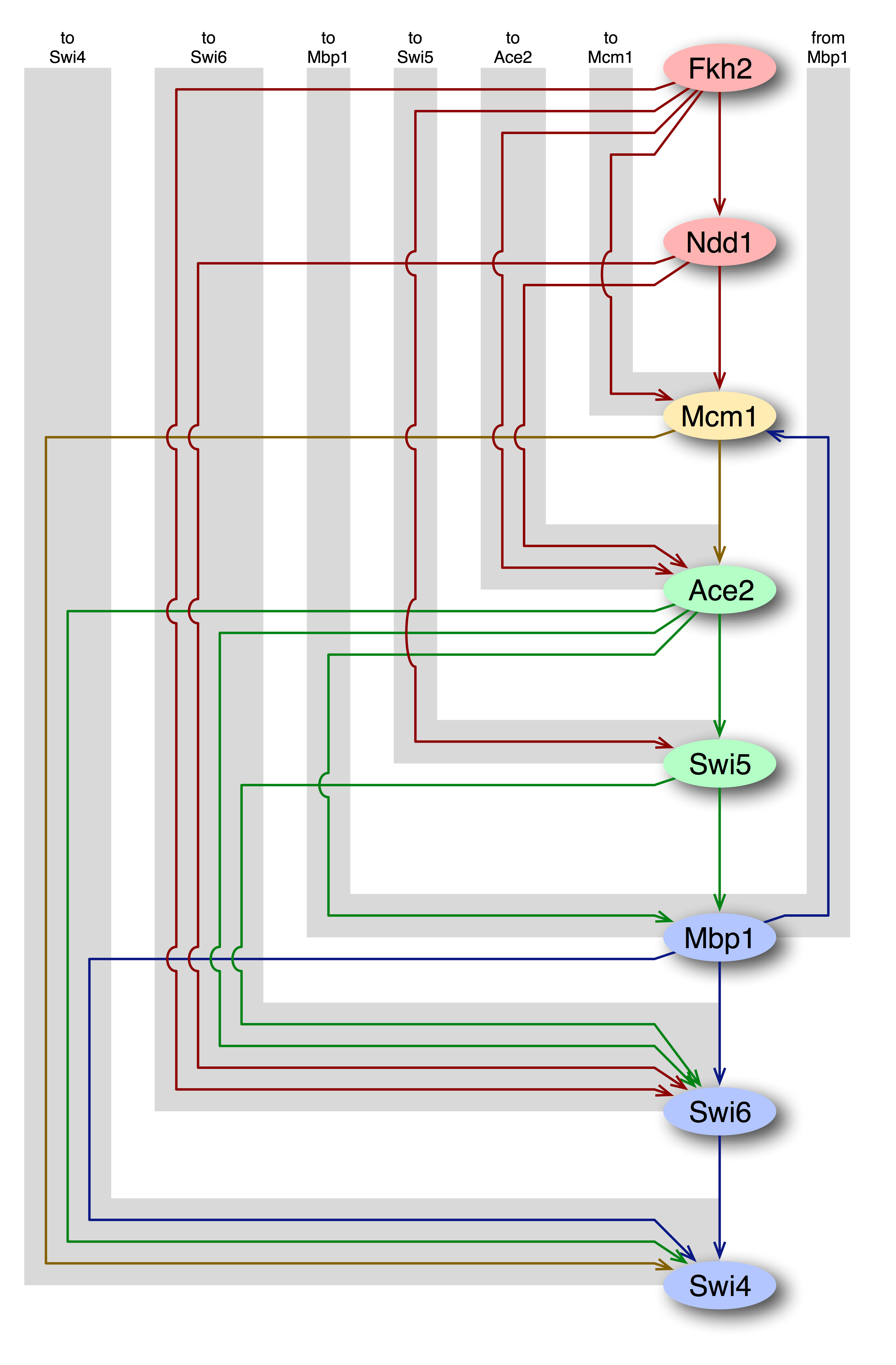

Supplement: Additional File 2 — Transcription factor network of canonical cell cycle regulators as derived from the elutriation time course. We show the non-zero entries in the model's time-translation matrix as directed arcs between transcription factors. We note the general similarity of the causal flow in this network to Figure 3 which was derived from the mating pheromone arrest time course. The order of factors displayed here minimizes the number of upward arcs (these arcs being grouped on the right side). [file 1471-2105-7-381-S2.tiff]

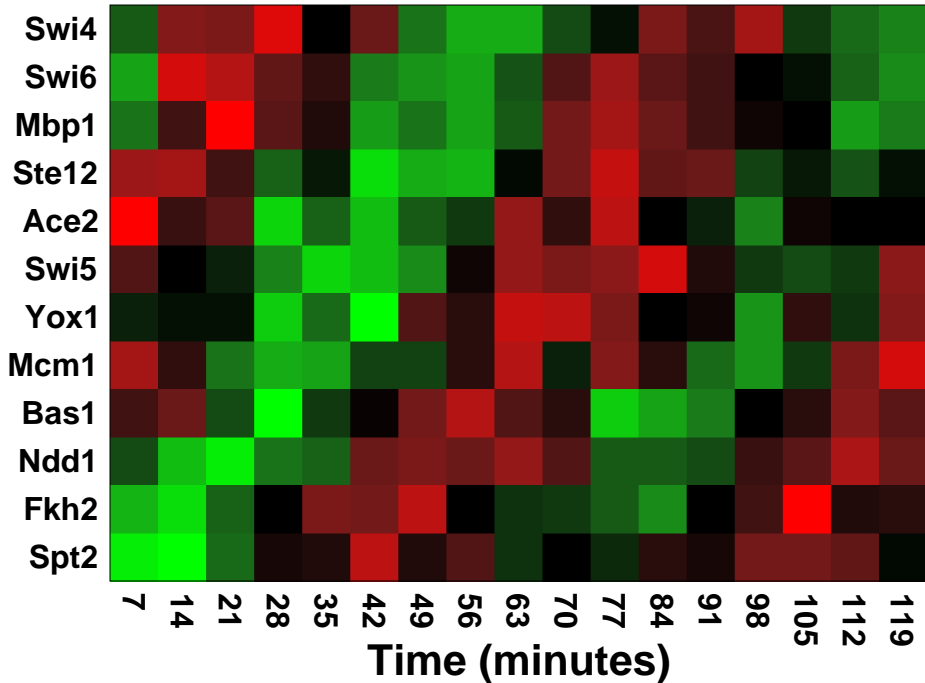

Supplement: Additional File 3 — α-coefficients of transcription factors regulating the cell cycle. A heat map (in the style of Figure 2) of the α-coefficients of the transcription factors from our extended 12-factor model during two periods of the cell cycle. [file 1471-2105-7-381-S3.pdf]

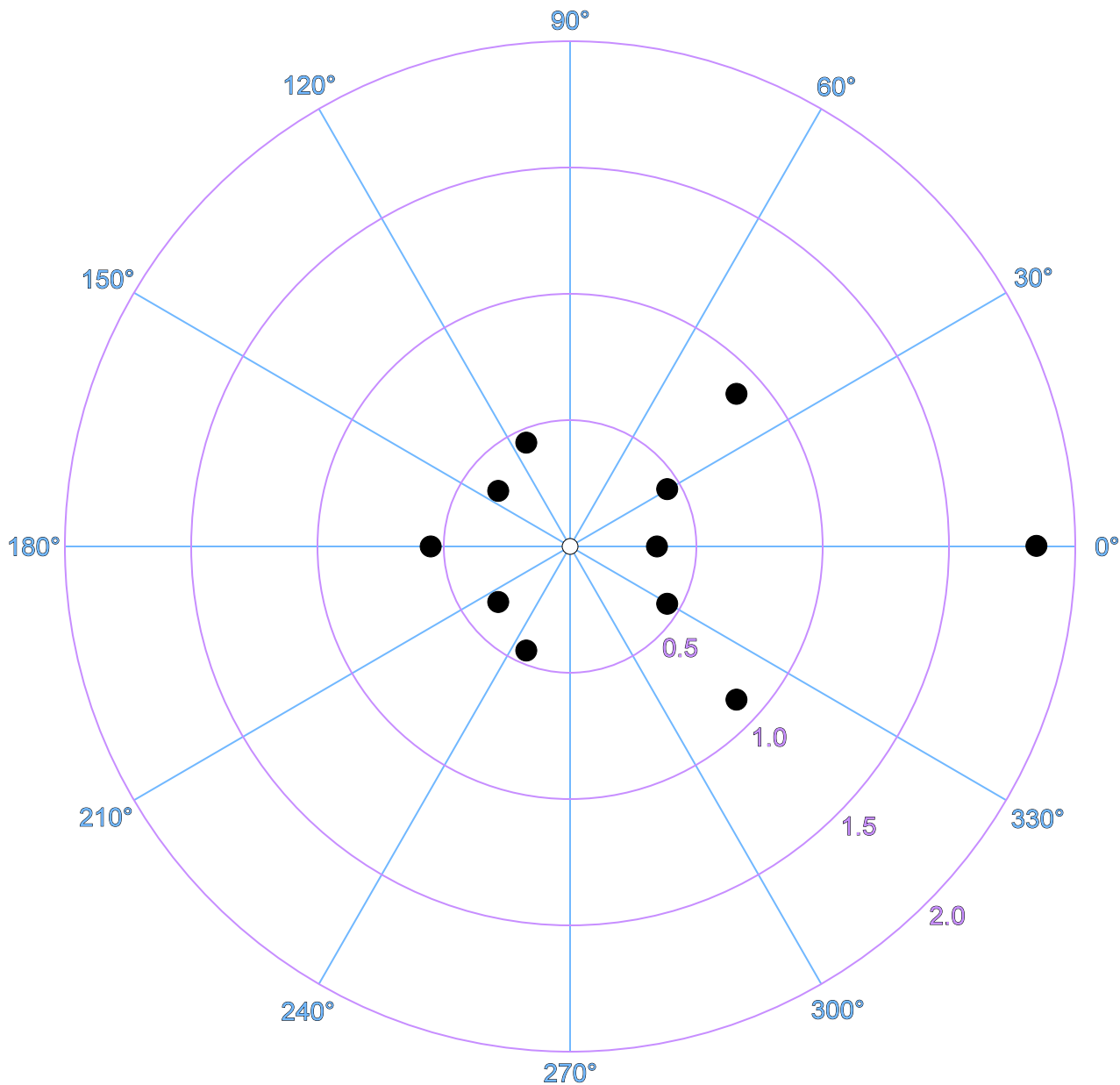

Supplement: Additional File 5 — Eigenvalues of the time-translation matrix of the 12-factor model. Each application of the time-translation matrix moves time forward 7 minutes. [file 1471-2105-7-381-S5.pdf]
