# Supplementary material for: Modelling the network of cell cycle transcription factors in the yeast Saccharomyces cerevisiae
Source: BMC Bioinformatics. 2006 Aug 16;7:381. doi: 10.1186/1471-2105-7-381 (PMC1570153; doi:10.1186/1471-2105-7-381)
Supplement: Additional File 4 — Transcription factor rankings used to select the 4 non-canonical transcription factors. Factors were ranked based on the number of time points in which their p-values were significant beyond some threshold (p < 0.1, 0.01, or 0.001). They were further ranked based on the periodicity of their α-coefficients. Canonical factors are shown in red, significant non-canonical factors are shown in yellow, and other factors are shown in white. We see that the same set of 5 non-canonical factors is selected as most significant for all of the p-value thresholds. [file 1471-2105-7-381-S4.pdf]

TF name	*Periodicity*	*Significant time points*	*Color*
p = 0.1			
Ndd1	0.300523	14	red
Swi6	0.315040	13	red
Swi5	0.204111	13	red
Swi4	0.327208	12	red
Mbp1	0.297817	12	red
Ace2	0.294236	10	red
Bas1	0.209138	10	yellow
Ste12	0.262012	9	yellow
Spt2	0.240477	9	yellow
Yox1	0.193717	9	yellow
Fkh1	0.165331	9	yellow
Abf1	0.090864	9	white
Msn4	0.082289	9	white
Mcm1	0.269384	7	red
Fkh2	0.256734	6	red
p = 0.01			
Ndd1	0.298176	14	red
Swi6	0.322294	13	red
Swi5	0.221195	13	red
Swi4	0.318195	12	red
Mbp1	0.284134	12	red
Ace2	0.298886	10	red
Bas1	0.191755	10	yellow
Fkh1	0.229689	9	yellow
Yox1	0.177898	9	yellow
Msn4	0.076258	9	white
Ste12	0.234691	8	yellow
Spt2	0.231186	8	yellow
Yap5	0.198903	8	white
Abf1	0.104303	8	white
Fhl1	0.102329	8	white
Mcm1	0.272602	7	red
Fkh2	0.260023	6	red
p = 0.001			
Ndd1	0.298176	14	red
Swi6	0.322294	13	red
Swi5	0.221195	13	red
Swi4	0.318195	12	red
Mbp1	0.284134	11	red
Ace2	0.298886	10	red
Bas1	0.191755	10	yellow
Fkh1	0.229689	9	yellow
Yox1	0.177898	9	yellow
Ste12	0.234691	8	yellow
Spt2	0.231186	8	yellow
Yap5	0.198903	8	white
Abf1	0.104303	8	white
Fhl1	0.102329	8	white
Msn4	0.076258	8	white
Mcm1	0.272602	7	red
Fkh2	0.260023	6	red
