# Supplementary material for: Modelling the network of cell cycle transcription factors in the yeast Saccharomyces cerevisiae
Source: BMC Bioinformatics. 2006 Aug 16;7:381. doi: 10.1186/1471-2105-7-381 (PMC1570153; doi:10.1186/1471-2105-7-381)
Supplement: Additional File 6 — Asymptotic phases and amplitudes. A derivation of the formulas used for the asymptotic phases and amplitudes of components of real systems governed by a general class of real time-translation matrices is given. [file 1471-2105-7-381-S6.pdf]

Supplementary material

Let A be an $n \times n$ real matrix and suppose A has not necessarily distinct eigenvalues $\lambda_1, \ldots, \lambda_n \in \mathbb{C}$ with $1 = |\lambda_1| = |\lambda_2| > |\lambda_3| \geq \cdots \geq |\lambda_n|$ and $\mathbb{C}$-linearly independent associated eigenvectors $v_1, \ldots, v_n \in \mathbb{C}^n \setminus \mathbf{0}$. It is a fact that the non-real eigenvalues come in complex conjugate pairs and, further, we may assume without loss of generality that the eigenvectors associated to such a pair are themselves conjugates. Suppose λ_1 and λ_2 are non-real complex conjugates and write $\lambda_1 = e^{i\theta}$, $\lambda_2 = e^{-i\theta}$ with $\theta \in \mathbb{R} \setminus \pi\mathbb{Z}$.

Given initial conditions $t_0 \in \mathbb{R}^n$, we have the time evolution $t_{k+1} := At_k \in \mathbb{R}^n$ for all $k \in \mathbb{N}$. We are interested in the asymptotic behavior of t_k for large k. Write V for the $n \times n$ complex matrix whose successive columns are $v_1, \ldots, v_n$. It can be shown that the rows $w_1, \ldots, w_n \in \mathbb{C}^n$ of $W := V^{-1}$ corresponding to conjugate v_i are themselves conjugates.

For all $k \in \mathbb{N}$,

$$
\begin{aligned}
t_k &= A^k t_0 = \left(V \begin{bmatrix} \lambda_1 & & \mathbf{0} \\ & \ddots & \\ \mathbf{0} & & \lambda_n \end{bmatrix} W \right)^k t_0 = V \begin{bmatrix} \lambda_1^k & & \mathbf{0} \\ & \ddots & \\ \mathbf{0} & & \lambda_n^k \end{bmatrix} W t_0 = V \begin{bmatrix} \lambda_1^k & & & \mathbf{0} \\ & \lambda_2^k & & \\ & & 0 & \\ & & & \ddots \\ \mathbf{0} & & & 0 \end{bmatrix} W t_0 + O(|\lambda_3|^k) \\
&= [v_1 v_2] \begin{bmatrix} \lambda_1^k & 0 \\ 0 & \lambda_2^k \end{bmatrix} \begin{bmatrix} w_1^\top \\ w_2^\top \end{bmatrix} t_0 + O(|\lambda_3|^k) = e^{ik\theta}(w_1^\top t_0) v_1 + e^{-ik\theta}(w_2^\top t_0) v_2 + O(|\lambda_3|^k)
\end{aligned}
$$

so that for $j \in 1..n$,

$$
\begin{aligned}
(t_k)_j &= (v_1)_j (w_1^\top t_0) e^{ik\theta} + (v_2)_j (w_2^\top t_0) e^{-ik\theta} + O(|\lambda_3|^k) \\
&= (v_1)_j (w_1^\top t_0)(\cos k\theta + i \sin k\theta) + \overline{(v_1)_j (w_1^\top t_0)(\cos k\theta + i \sin k\theta)} + O(|\lambda_3|^k) \\
&= 2\operatorname{Re}\left((v_1)_j (w_1^\top t_0)(\cos k\theta + i \sin k\theta)\right) + O(|\lambda_3|^k) \\
&= 2\operatorname{Re}\left((v_1)_j (w_1^\top t_0)\right)\cos k\theta - 2\operatorname{Im}\left((v_1)_j (w_1^\top t_0)\right)\sin k\theta + O(|\lambda_3|^k).
\end{aligned}
$$

Note for $(\alpha, \beta) \in \mathbb{R} \setminus \mathbf{0}$ and $\phi \in \mathbb{R}$ that

$$
\begin{aligned}
\alpha \cos\phi - \beta \sin\phi &= \sqrt{\alpha^2 + \beta^2} \left(\frac{\alpha}{\sqrt{\alpha^2 + \beta^2}} \cos\phi - \frac{\beta}{\sqrt{\alpha^2 + \beta^2}} \sin\phi \right) \\
&= \sqrt{\alpha^2 + \beta^2} \left((\cos \arctan(\alpha, \beta)) \cos\phi - (\sin \arctan(\alpha, \beta)) \sin\phi \right) \\
&= \sqrt{\alpha^2 + \beta^2} \cos(\phi + \arctan(\alpha, \beta))
\end{aligned}
$$

and so $(t_k)_j = 2\left|(v_1)_j (w_1^\top t_0)\right| \cos\left(k\theta + \arg (v_1)_j (w_1^\top t_0)\right) + O(|\lambda_3|^k)$, assuming $(v_1)_j (w_1^\top t_0) \neq 0$ (otherwise $(t_k)_j = O(|\lambda_3|^k)$, or alternatively assign some arbitrary value to $\arg 0$). $\square$
